# Nanostructured Higher Manganese Silicide Thermoelectrics Developed by Mechanical Alloying Using High-Purity and Recycled Silicon

**DOI:** 10.3390/nano15161286

**Published:** 2025-08-21

**Authors:** Panagiotis Mangelis, Kostas Georgiou, Panagiotis Savva Ioannou, Savvas Hadjipanteli, Anne-Karin Søiland, Theodora Kyratsi

**Affiliations:** 1 Department of Mechanical and Manufacturing Engineering, University of Cyprus, Nicosia 1678, Cyprus; georgiou.kostas@ucy.ac.cy (K.G.); ioannou.s.panagiotis@ucy.ac.cy (P.S.I.); hadjipanteli.savvas@ucy.ac.cy (S.H.); 2 ReSiTec AS, Setesdalsveien 110, 4617 Kristiansand, Norway; aks@resitec.no

**Keywords:** higher manganese silicides, mechanical alloying, thermoelectric properties, hot-press sintering, recycled silicon kerf

## Abstract

Mechanical alloying (MA) has been proven to be an energy-efficient synthetic route for the development of high-performance thermoelectric (TE) materials. Higher Manganese Silicide (HMS) phases of the general formula Mn(Si_1−x_Al_x_)_1.75_ (0 ≤ x ≤ 0.05) were prepared by MA implementing a short-time ball-milling process. Powder XRD and SEM analysis were carried out to validate the HMS phases, while small amounts of the secondary phase, MnSi, were also identified, especially for the Al-doped products. Electrical transport properties measurements showed that Al substitution causes an effective hole doping. A remarkable increase in electrical conductivity is observed for the Al-doped phases, while the corresponding reduction in the Seebeck coefficient is indicative of the increase in carrier density. Despite the small percentages of MnSi detected in Al-doped phases, an improvement in TE efficiency is achieved in the series Mn(Si_1−x_Al_x_)_1.75_ (0 ≤ x ≤ 0.05). The 2.5% Al-doped phase exhibits a maximum figure-of-merit (ZT) of 0.43 at 773 K. Moreover, in an effort to utilize recycled silicon byproducts from photovoltaic (PV) manufacturing, Al-doped phases are developed by MA using two types of Si kerf. The two kerf-based products exhibit lower TE efficiencies, due to the increased amounts of the metallic MnSi phase.

## 1. Introduction

In recent years, the development of green energy technologies with zero-carbon emissions is an urgent matter due to climate change and the energy crisis. Thermoelectric (TE) devices are able to produce clean electricity through the Seebeck effect and to provide energy recovery by utilizing waste heat emitted to the environment [1,2]. Recent advancements on the discovery and development of more efficient, environmentally friendly and low-cost TE materials enhance the efforts for thermoelectrics to be established as a feasible sustainable technology in the coming years, overcoming challenges that prevent their present commercialization [2,3,4,5,6].

In addition, new environmental issues have arisen during the last few years that are related to the waste management and recycling of raw materials. The recycling and reuse of materials with high production cost and the establishment of a circular economy approach in high-technology sectors, such as the electronic and semiconductors industry, is currently one of the main environmental priorities [7,8]. The production of high-purity crystalline silicon wafers in the photovoltaic (PV) industry through Siemen’s method is an expensive and energy-costly procedure [9], while large amounts of Si kerf byproducts are wasted during the manufacturing process of PV panels [10]. The group of silicide thermoelectrics provides a great opportunity to open a new route for recycling vast amounts of waste materials and byproducts from the silicon industry [11]. So far, our research efforts on the utilization of recycled silicon based on Si kerf byproducts from PV manufacturing and the development of *n*-type silicides are quite promising [12,13,14,15]. On the other hand, research concerning *p*-type silicides developed by recycled silicon is very limited [16].

Higher Manganese Silicide (HMS) compounds with stoichiometry MnSi*_γ_* (1.73 < *γ* < 1.77) exhibit the most promising TE efficiencies from the silicides family. They are *p*-type degenerate semiconductors with good electrical conductivity and a relatively high Seebeck coefficient [17,18]. In addition, the HMS phases demonstrate a range of beneficial characteristics, since they consist of earth-abundant, low-cost, and environmentally friendly elements [19]. They also satisfy other important parameters, critical in the fabrication of TE devices for medium-temperature applications, such as sufficient thermal and chemical stability [20]. Structural investigations have shown that HMS compounds belong to the Nowotny chimney ladder (NCL) phases [21,22]. Mn sub-lattice forms a tetragonal unit cell that is interpenetrated by Si atoms, creating a helical ladder. The NCL tetragonal phases present similar values for the *a* lattice constant, while the perpendicular *c* constant, which is dependent on the Si sub-lattice, exhibits high variations in nanometers. The NCL phases usually coexist, while the existence of MnSi as a secondary phase is quite common due to a peritectic reaction that takes place between MnSi and liquid for the formation of MnSi_γ_ [19,23]. Recent studies have shown the incommensurate nature of HMS phases due to the silicon modulation along the perpendicular axis, which is described more precisely by using a (3+1)-dimensional superspace group approach [24,25,26,27]. 

Several reports have shown that the optimization of TE properties can be accomplished by implementing hole doping. The substitution of small amounts of silicon or manganese with appropriate dopants results in the increase in hole density, leading to Power Factor (PF) and figure-of-merit (ZT) improvements [28,29,30,31,32,33,34]. The substitution of Si by Al is a very promising strategy to enhance the TE efficiency of HMS phases [27,35,36]. In addition, chemical substitution with isoelectronic atoms have been attempted, targeted toward the reduction in thermal conductivity [37,38,39]. It is notable that supersaturated Re substitution at the Mn site has driven to high ZT improvements due to the significant decrease in lattice thermal conductivity [40,41,42]. Several attempts have also been carried out to exploit synergies for the enhancement in TE performance of HMS phases by implementing multiple substitution, combining different dopants, or introducing appropriate nanoinclusions, with the aim of optimizing the electrical transport properties and promoting effective phonon scattering [33,37,43,44,45,46,47,48,49]. Moreover, due to the hard and brittle nature of HMS, efforts have also been performed to improve its mechanical response, especially in application environments with large temperature gradients and thermal shocks. The secondary phase, MnSi, has a strong impact on the failure mechanism of the material, since its existence in small amounts results in many microcracks that weaken its mechanical behavior [50]. Various strategies, including vanadium addition and consolidation methods, also very strongly influence the mechanical properties of HMS [51].

The synthetic route that is followed in the development of HMS phases plays an important role in their TE properties. A wide range of methods have been studied until now, accomplishing different efficiencies for the pristine and doped phases. Several high-temperature methods have been performed, such as arc melting [28,33,52,53], induction melting [54,55], melt-quenching [56], and melt-spinning [35,40,57,58], which have been used to develop HMS compounds with the non-doped phases to exhibit ZT values between 0.4 and 0.55 [19]. However, these methods require high amounts of energy, increasing the production cost; moreover, they are not appropriate to scale-up the synthesis process. On the other hand, MA is quite attractive since it is an energy-efficient and simple method. In addition, this technique is favorable for the development of nanostructured phases, which enhance phonon scattering due to the increase in grain boundaries. Comparing ball milling with arc melting and melt-spinning, an appreciable reduction in lattice thermal conductivity was observed due to the nanostructuring effect [59]. Several studies have performed ball milling for the synthesis of HMS phases [60,61,62,63,64] or the development of nanocomposites [65]. The existence of MnSi in small amounts is quite common in the MA-developed phases since a mechanochemical decomposition takes place during ball milling [61,64]. This decomposition seems to come from the asymmetrical structural features of NCL phases and is closely related to the grain size of nanostructured materials [61,66]. MnSi is a metallic phase [67], which has a negative impact on the TE efficiency of HMS. It affects the carrier density of the system, inducing a severe decline in the Seebeck coefficient, while simultaneously it increases the lattice thermal conductivity, as previous reports have shown [68,69]. Studies have shown that the optimization of ball-milling parameters [61] or the usage of organic solvents as milling media [61,70] can restrict MnSi.

In this study, Al-doped HMS phases with the general formula Mn(Si_1−x_Al_x_)_1.75_ (0 ≤ x ≤ 0.05) are developed by MA and hot-press sintering. It is the first time in which hole doping through Al substitution is systematically studied in the MA-based MnSi_1.75_ system, investigating in parallel the effect of the ball-milling process in the TE performance of the developed materials. Powder X-ray diffraction (XRD), scanning electron microscopy (SEM) and energy dispersive X-ray spectroscopy (EDX) are performed for the characterization of Mn(Si_1−x_Al_x_)_1.75_ phases. An effective hole doping is achieved through Al substitution, increasing the carrier density and optimizing the TE properties across the series Mn(Si_1−x_Al_x_)_1.75_ (0 ≤ x ≤ 0.05). In addition, the development of Al-doped phases by using recycled silicon, based on byproducts from PV industry, is also attempted in this investigation. In an effort to provide a sustainable solution in the recycling of silicon, high-purity silicon is replaced by two types of recycled silicon from Si kerf for the development of *p*-type silicide thermoelectrics.

## 2. Materials and Methods

Mn(Si_1−x_Al_x_)_1.75_ (0 ≤ x ≤ 0.05) phases were prepared by the MA method using commercially available high-purity reagent elements, Mn, Al, and Si (>99.9%, Alfa Aesar, Haverhill, MA, USA) and recycled silicon based on Si kerf from the PV industry. Mixing of the reagent materials with appropriate stoichiometry was carried out in a glovebox and ball milling took place in a tungsten carbide vial under an argon atmosphere using a planetary mill (Pulverisette 6, Fritsch, Idar-Oberstein, Germany) and implementing a ball-to-powder ratio (BPR) of 20:1 with a speed of 450 rpm for 6 h. Hot-press sintering was carried out using a hot-press HP20 (Thermal Technologies LLC, Bedford, NH, USA) for the densification of the nanopowders into pellets under an argon atmosphere at 1030 °C with a pressure of 80 MPa and a heating rate of 10 °C/min for 1 h.

Powder XRD was performed for the characterization of the phases by using a SmartLab diffractometer (Rigaku, Tokyo, Japan) (45 kV, 200 mA) with Cu-Kα radiation and setting a scan step of 0.02° over the range 20 ≤ 2θ/° ≤ 90 with a time per step of 0.6 s. Rietveld refinements were carried out using the GSAS-I software package. SEM imaging was performed on the powder and polished pellets for the study of nano- and microstructure morphology of the developed materials using an electron microscope Vega II LSU (Tescan, Brno, Czech Republic) of 20 kV and a backscattered electron (BSE) detector. A Vega II LSU instrument together with an EDX detector (Princeton Gamma Tech, Princeton, NJ, USA) was used for EDX measurements. EDX spectra were collected by multiple scans with a 45° take-off angle and 100 s live time. The ZAF matrix correction quantification protocol was used for the elemental analysis and the calculation of atomic percentages.

The density of fabricated pellets was determined geometrically. Electrical conductivity (σ) and Seebeck coefficient (S) measurements were performed in the temperature range 300 K ≤ T ≤ 773 K using a ZEM3 system (ULVAC – RIKO, Yokohama, Japan). A LFA 457 laser instrument (Netzsch, Selb, Germany) was used for thermal diffusivity (D) measurements on pellets coated with graphite. The heat capacity (*C_p_*) was determined with a pyroceram reference and the thermal conductivity was calculated using the formula $\kappa =D\rho C_{p}$. The uncertainties for the aforementioned electrical and thermal transport property measurements are ±5% and ±7%, respectively.

## 3. Results

Nanostructured HMS phases were synthesized by the MA method using high-purity silicon. The developed materials were characterized by powder XRD, SEM, and EDX. Al doping was performed in order to increase carrier concentration and optimize the TE properties of the series Mn(Si_1−x_Al_x_)_1.75_ (0 ≤ x ≤ 0.05). Two types of recycled Si based on Si kerf were also used for the replacement of high-purity silicon and the development of Al-doped phases with the optimum stoichiometry and best TE properties.

### 3.1. Mn(Si_1−x_Al_x_)_1.75_ (0 ≤ x ≤ 0.05) Phases Developed Using High-Purity Silicon

**Figure 1:**
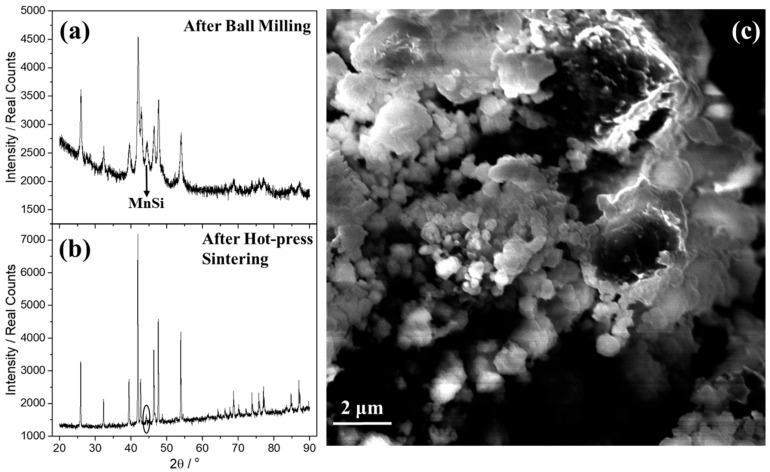
Powder XRD for the MnSi_1.75_ phase (**a**) after the ball-milling process and (**b**) after hot-press sintering. (**c**) SEM image of the nanostructured powder of MnSi_1.75_ phase after ball milling.

Initially, MA was carried out for the development of the pristine phase, MnSi_1.75_. Powder XRD was carried out to study the purity of produced phase and to identify possible secondary phases before and after hot-press sintering (Figure 1a,b). It is obvious from the two patterns that the crystallinity of material increased after hot-press sintering. The characteristic broadening of the Bragg peaks after ball milling denotes a lower average crystallite size than that after hot-press sintering, in which the peaks become narrow and long. Using the Scherrer equation, it is confirmed that the average crystallite size increased from 17.6 nm (after ball milling) to 84.0 nm after hot-press sintering. In addition, hot-press sintering favors the suppression of the secondary phase, MnSi, since there is a reduction in intensity of its characteristic reflection, close to 44.3°, after hot-press sintering. After performing ball milling for different times (3, 6, and 9 h), powder XRD data after hot-press sintering (Figure S1a in Supplementary Materials) show that MnSi_1.75_ exhibits almost a single phase with only slight traces of MnSi identified for all investigated ball-milling times. However, in Figure S1b it is obvious that for ball milling 3 and 6 h, the characteristic peak of MnSi remains at the same levels, while for 9 h a slight increase is observed. For this reason, ball milling for 6 h was selected later for the development of the Al-doped phases. SEM analysis was also performed on the nanostructured powder of the pristine phase in order to examine results concerning the grain morphology. As can be observed in Figure 1c, the distribution of grain size ranges between ca. 2 μm and 400 nm, with the small nanoparticles exhibiting a spherical shape. 

**Figure 2:**
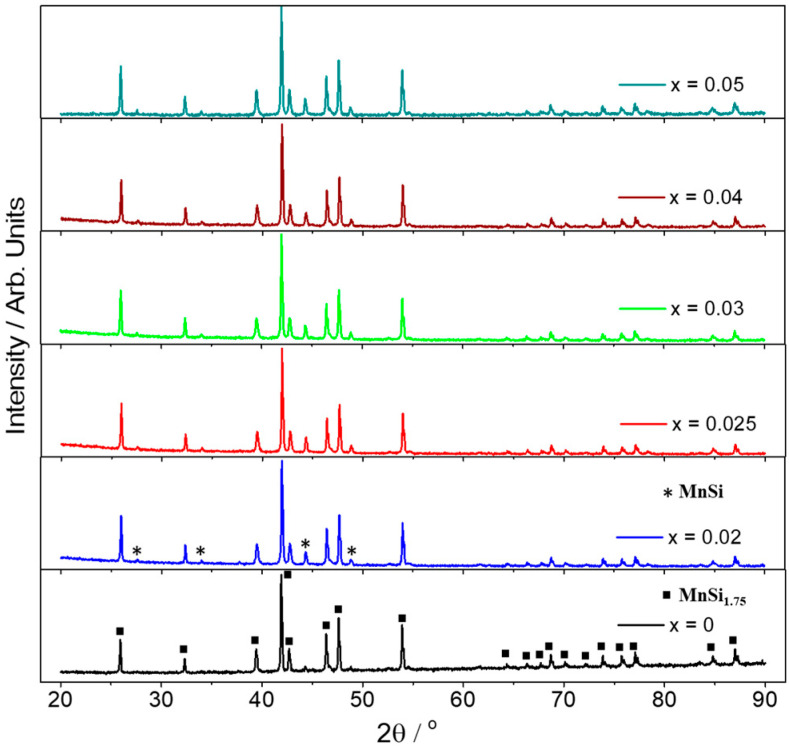
Powder XRD patterns of the products in the series Mn(Si_1−x_Al_x_)_1.75_ (0 ≤ x ≤ 0.05) after hot-press sintering. Reflection positions for the HMS phase, MnSi_1.75_, and the secondary phase, MnSi, are marked with squares and asterisks, respectively.

The development of Al-doped–HMS phases followed, implementing the same ball-milling conditions across the series Mn(Si_1−x_Al_x_)_1.75_ (0 ≤ x ≤ 0.05). Figure 2 presents powder XRD data after hot-press sintering, validating the formation of the HMS phase for all the materials. However, a small increase in MnSi phase is observed in all Al-doped products in comparison with the pristine phase. Backscattering SEM analysis was carried out on the surface of pellets to study the microstructure of the sintered materials. 

**Figure 3:**
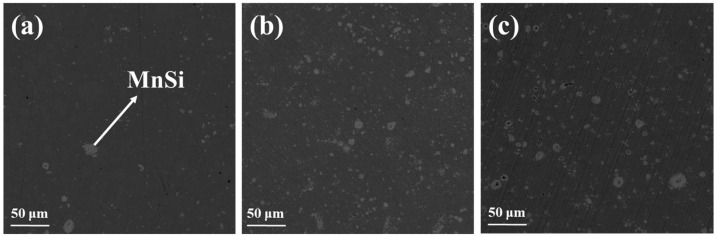
Backscattering SEM images of the Mn(Si_1−x_Al_x_)_1.75_ (0 ≤ x ≤ 0.05) phases with (**a**) x = 0, (**b**) x = 0.025, and (**c**) x = 0.05.

As can be observed in Figure 3, there is a noticeable difference between the images of the pristine phase and those of the Al-doped phases. The dark matrix denotes the HMS phase, while the light gray spots are indicative of the MnSi secondary phase. It is obvious that the number of spots is larger in the two Al-doped phases, confirming the results of powder XRD. EDX measurements were also performed using the pristine phase MnSi_1.75_, as can be observed in Figure S2. The elemental analysis validates that the dark gray matrix corresponds to the HMS phase, while the light gray spots depict the secondary phase, MnSi.

**Figure 4:**
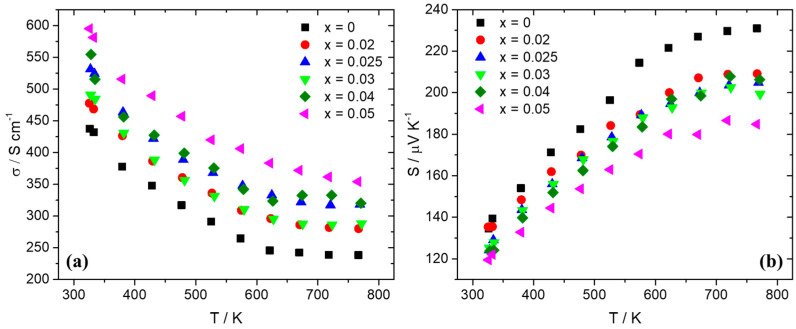
(**a**) Electrical conductivity and (**b**) Seebeck coefficient data for Mn(Si_1−x_Al_x_)_1.75_ (0 ≤ x ≤ 0.05) as a function of temperature.

**Figure 5:**
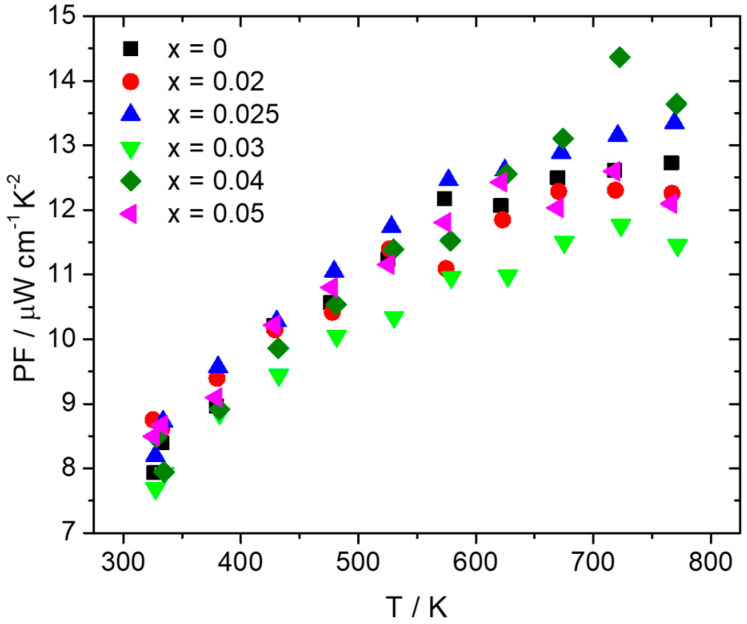
Power factor of the Mn(Si_1−x_Al_x_)_1.75_ (0 ≤ x ≤ 0.05) series as a function of temperature.

Thermoelectric property measurements followed after the consolidation of pellets through hot-press sintering, reaching densities that were more than 95% of theoretical density. Figure 4 presents electrical conductivity and the Seebeck coefficient data for the series Mn(Si_1−x_Al_x_)_1.75_ (0 ≤ x ≤ 0.05). As can be observed, there is an increase in electrical conductivity for the Al-doped phases compared with the pristine material. The sample with the highest Al doping level of 5% exhibits the maximum values of electrical conductivity. In Figure 3b, the Seebeck coefficient data present positive values, indicative of *p*-type behavior. The reduction in S with the increase in Al content across the series Mn(Si_1−x_Al_x_)_1.75_ (0 ≤ x ≤ 0.05) denotes an increase in hole concentration. The increase in hole concentration is also the reason for the rise in electrical conductivity. As shown in Figure 5, hole doping through Al substitution results in an improvement in PF with the Al 2.5 and 4% phases presenting the highest values. The increase in electrical conductivity prevails over the reduction in the Seebeck coefficient. A maximum PF of ca. 14.5 μW cm^−1^ K^−2^ is exhibited at 725 K by the 4% Al-doped phase.

**Figure 6:**
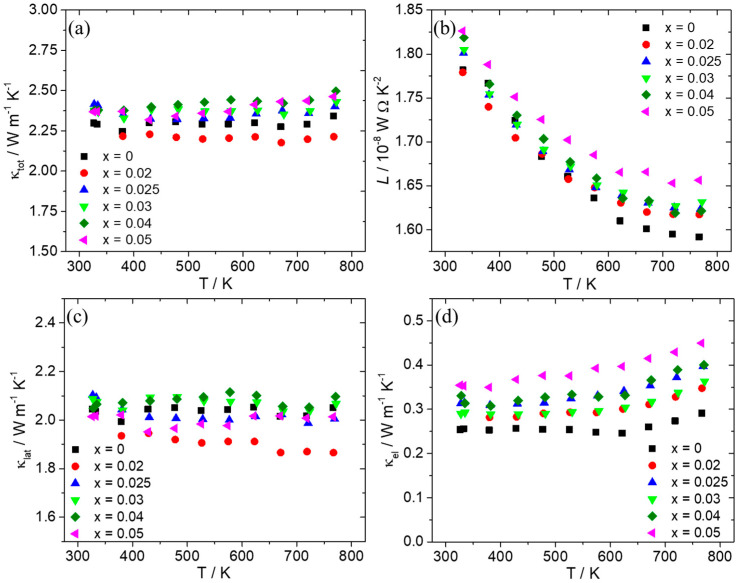
(**a**) Total thermal conductivity (κ_tot_), (**b**) Lorenz number (*L*), (**c**) lattice thermal conductivity (κ_lat_), and (**d**) electronic thermal conductivity (κ_el_) of the Mn(Si_1−x_Al_x_)_1.75_ (0 ≤ x ≤ 0.05) series as a function of temperature.

Figure 6 shows thermal conductivity data and the contributions from lattice (κ_lat_) and charge carriers (κ_el_) as calculated using the formula for the Wiedemann–Franz law: κ_el_ = *L*.σ.Τ. Taking into account scattering from acoustic phonons [71,72], the Lorenz number (*L*) was determined from the Seebeck coefficient using Fermi–Dirac statistics (Figure 6b). As can be observed, the Al-doped phases exhibit total thermal conductivity values close to those of the pristine material, with a slight increase observed for x ≥ 0.025. This seems to come mainly from the increase in electronic contribution since the lattice contribution does not show noticeable changes for those samples. However, it must be noted that for x = 0.02, a favorable reduction in lattice thermal conductivity is observed, resulting in a decrease in total thermal conductivity in comparison with the pristine material.

**Figure 7:**
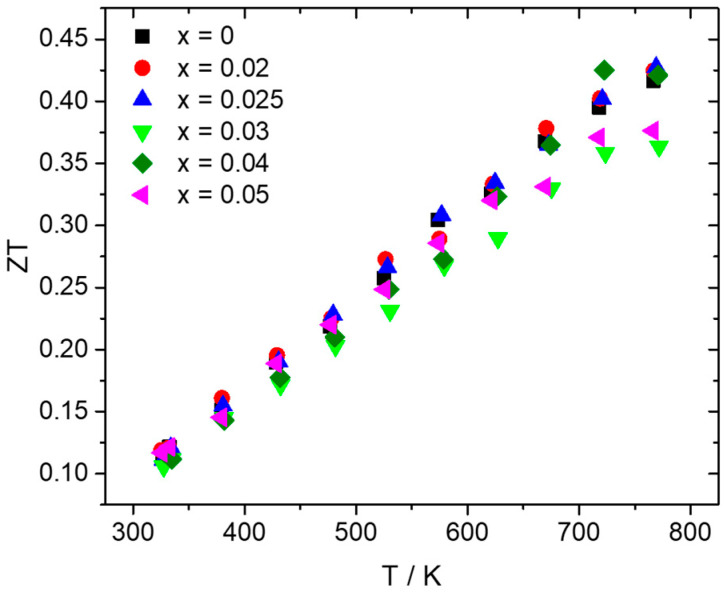
TE figure-of-merit ZT for the Mn(Si_1−x_Al_x_)_1.75_ (0 ≤ x ≤ 0.05) series as a function of temperature.

Figure 7 presents the TE figure-of-merit ZT as a function of temperature, as determined by the aforementioned property measurements. Most of the Al-doped phases present ZT values at the same levels with those of the pristine material along the whole temperature range, with a slight increase observed for x = 0.025 and x = 0.04 at higher temperatures due to the improvement in PF. The Al-doped phase with x = 0.025 exhibits the maximum value of 0.43 at 773 K.

### 3.2. Mn(Si_0.975_Al_0.025_)_1.75_ Phases Developed Using Recycled Silicon Kerf

In an effort to reuse recycled silicon derived from byproducts from industrial processes of PV manufacturing, recycled Si kerf was used for the synthesis of Mn(Si_0.975_Al_0.025_)_1.75_ phases by MA. The Si kerf is a byproduct from the sawing of wafers. To produce the recycled silicon from Si kerf, low-energy intensive processing routes are employed, yielding substantially lower production cost and carbon footprint than commercial silicon powders. Two purification routes, RST 1-2 and RST ODIN-0821, differ mainly in their efficiency for the removal of aluminum and carbon.

Results about the purity of recycled silicon from Si kerf have been reported in detail in our previous study, showing slight traces of impurities such as Al, Ca, and Ni in the order of a few hundred ppm [13]. Table S1 presents the exact amounts of elemental impurities for the two recycled silicon kerf byproducts. Performing STEM-EELS measurements, a silicon oxide layer of 1−2 nm was also identified on the surface of Si nanoparticles of both kerfs. However, it must be noted that the RST 1-2 kerf presents slightly higher levels of purity than that of RST ODIN-0821 because a different purification method was used. The same MA conditions were applied and an Al content of 2.5% was added since this amount of doping provided the best TE performance, as shown previously. 

**Figure 8:**
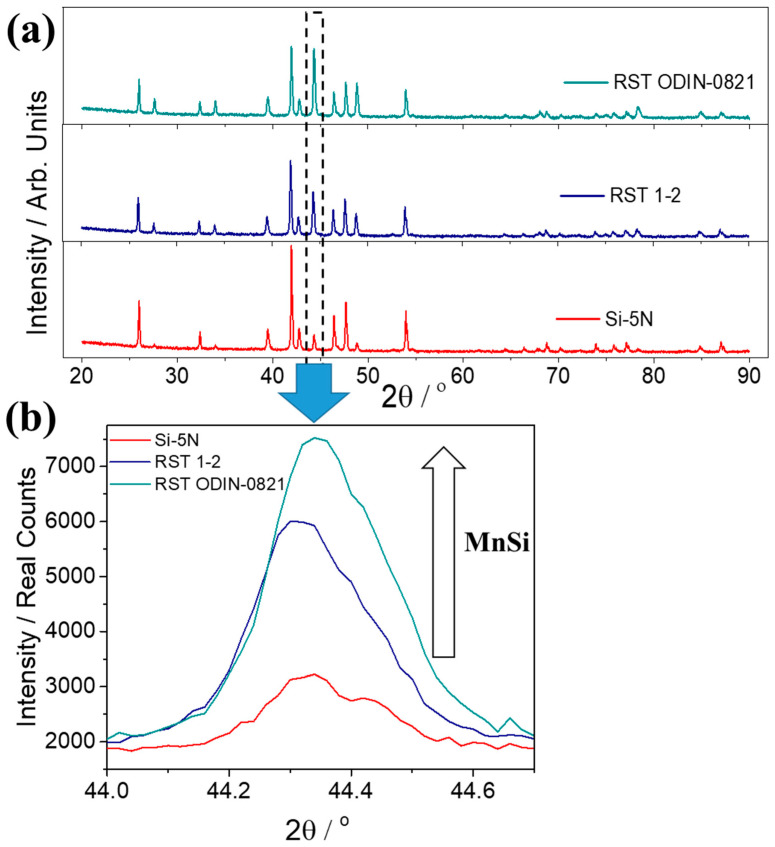
(**a**) Powder XRD patterns of the Mn(Si_0.975_Al_0.025_)_1.75_ phases based on Si-5N and two Si kerfs, RST 1-2 and RST ODIN-0821, in the angular range 20 ≤ 2θ/° ≤ 90. (**b**) The characteristic Bragg reflection of secondary phase, MnSi, close to 44.3°.

Figure 8a presents powder XRD data for the two kerf-based phases in comparison with that of the Si-5N-based counterpart. The patterns of the two products developed by the two types of recycled silicon from Si kerf confirm the formation of the desired HMS phase. However, as shown in Figure 8b, it is clearly obvious that the amount of the secondary phase, MnSi, presents an appreciable increase in the two kerf cases. Comparing the two recycled silicon-based phases, the RST ODIN-0821 case exhibits higher percentages of the MnSi phase. Rietveld refinements were performed on the 2.5% Al-doped products based on the high-purity silicon and the two types of Si kerf (Figure S3 in Supplementary Materials). The refinements showed that the Mn_15_Si_26_ superstructure provides a better description of the developed HMS phases than Mn_4_Si_7_, while the existence of MnSi is also validated as a secondary phase in all of the powders. The results from the quantification analysis (Table S2 in Supplementary Materials) confirm the increased levels of MnSi for the two kerf cases, with the RST ODIN-0821 exhibiting about 38 wt.% of the metallic phase. The existence of MnSi in such large amounts will have a strong impact in the TE properties of the materials.

**Figure 9:**
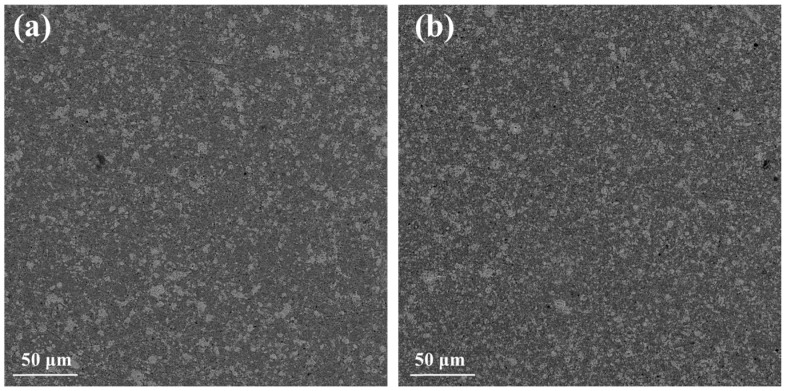
Backscattering SEM images of Mn(Si_0.975_Al_0.025_)_1.75_ based on (**a**) RST 1-2 and (**b**) RSTODIN-0821 Si kerfs.

Figure 9 presents backscattering SEM images for the two recycled silicon cases. The differences with those of the Si-5N cases shown in Figure 3 are noticeable. The large number of light gray spots in the microstructure of the two recycled silicon cases indicates the formation of MnSi in much larger amounts compared with those of the high-purity Si cases. In addition, the images of the RST ODIN-0821 case present a denser mosaic-like microstructure in comparison with that of RST 1-2, confirming the increased percentages of MnSi reported in the powder XRD data.

**Figure 10:**
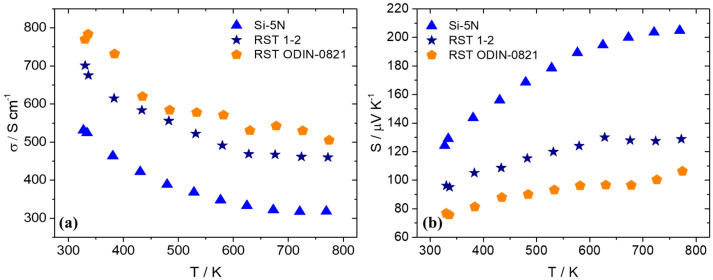
(**a**) Electrical conductivity and (**b**) Seebeck coefficient data of Mn(Si_0.975_Al_0.025_)_1.75_ phases based on Si-5N, RST 1-2, and RSTODIN-0821, as a function of temperature.

**Figure 11:**
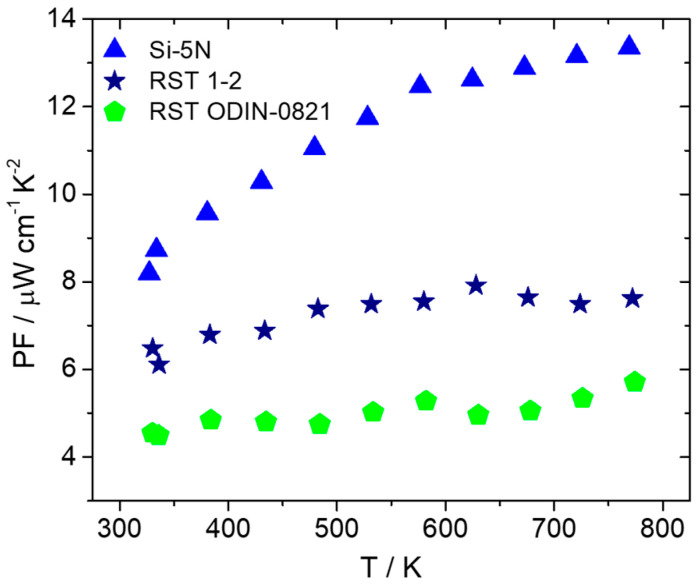
PF of Mn(Si_0.975_Al_0.025_)_1.75_ phases based on Si-5N, RST 1-2, and RSTODIN-0821, as a function of temperature.

After the fabrication of pellets implementing hot-press sintering, as previously described, electrical and thermal transport property measurements were carried out. Figure 10 presents electrical conductivity and the Seebeck coefficient data for the two recycled silicon cases in comparison with the high-purity Si phase. As shown, there is an obvious increase in electrical conductivity for the recycled-based materials with the RST ODIN-0821 case, which presents slightly higher values than those of the RST 1-2 counterpart. Simultaneously, an appreciable decrease in the Seebeck coefficient for the two recycled silicon cases implies an increase in carrier density for the two kerf-based systems. As a result, the PF is reduced for the two recycled silicon phases, as shown in Figure 11. However, the RST 1-2 case exhibits better values than those for RST ODIN-0821 due to the improved Seebeck coefficient, reaching a maximum PF of 7.9 μW cm^−1^ K^−2^ at 627 K.

**Figure 12:**
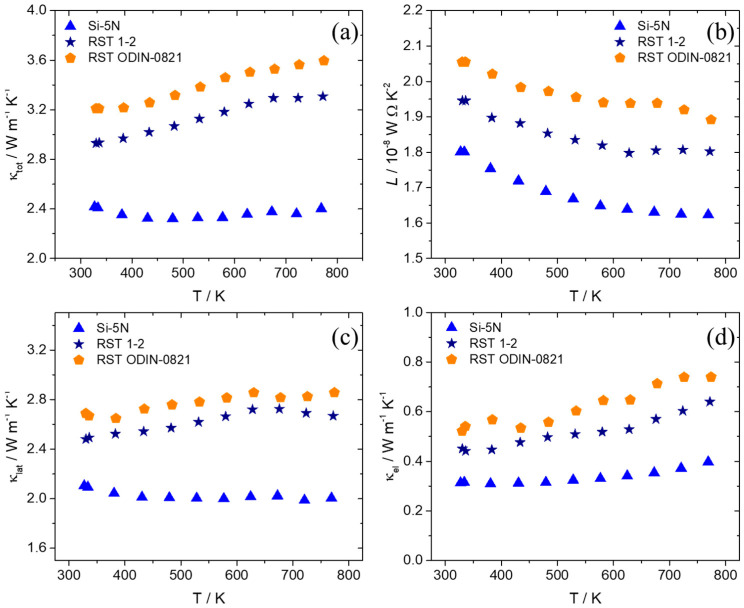
(**a**) Total thermal conductivity (κ_tot_), (**b**) Lorenz number (*L*), (**c**) lattice thermal conductivity (κ_lat_), and (**d**) electronic thermal conductivity (κ_el_) of the Mn(Si_0.975_Al_0.025_)_1.75_ phases based on Si-5N, RST 1-2, and RSTODIN-0821, as a function of temperature.

Thermal conductivity measurements are presented in Figure 12 for the recycled silicon-based products in comparison with those of their Si-5N counterpart. The electronic and lattice contributions are determined using the Wiedemann–Franz law, and the Lorenz number is calculated as previously described. An appreciable increase in total thermal conductivity is observed for the two recycled silicon cases, with the RST ODIN-0821-based product presenting the maximum values across the temperature range. Both lattice and electronic thermal conductivities contribute positively to this increase, with κ_lat_ affecting this more strongly than κ_tot_. The Lorenz number is also increased for the two kerf-based systems, indicating higher levels of carrier concentration and reaching values closer to the degenerate limit [73]. 

**Figure 13:**
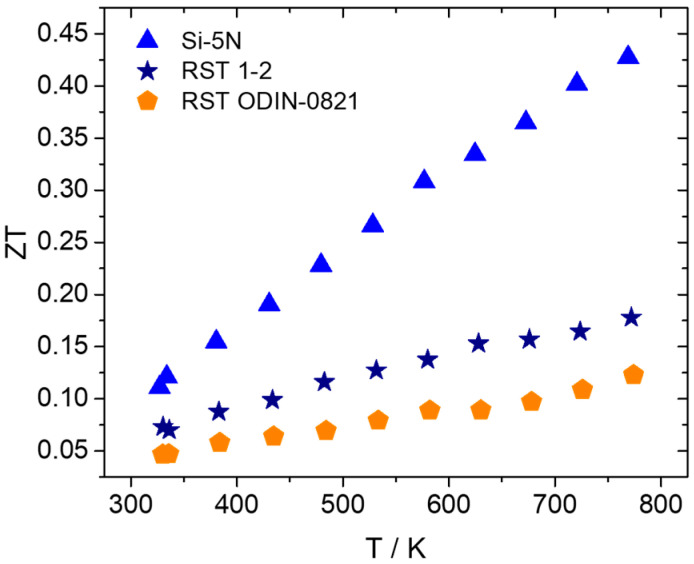
TE figure-of-merit ZT of Mn(Si_0.975_Al_0.025_)_1.75_ phases based on Si-5N, RST 1-2, and RSTODIN-0821, as a function of temperature.

Based on the aforementioned electrical and thermal transport property measurements, the TE efficiency, ZT, was calculated for the kerf-based products. Figure 13 presents ZT values for the two recycled silicon cases in comparison with those of the Si-5N phase. As expected, a reduction in ZT is observed for the two recycled-based systems, compared with the high-purity Si phase. The reduced PF and simultaneous increased values of total thermal conductivity affect negatively the efficiency of the recycled-based systems. Comparing the two recycled silicon cases, it is obvious that RST 1-2 overcomes RST ODIN-0821 in terms of TE properties and performance, reaching a maximum ZT of 0.18 at 773 K.

## 4. Discussion

The dry MA conditions and hot-press sintering applied initially for the development of the pristine compound produced successfully the desired HMS phase, apart from slight traces of MnSi precipitates identified by powder XRD, backscattering SEM imaging, and EDX analysis. In addition, powder XRD shows that the production of final phase with MA is not straightforward and that the heat treatment applied during hot-press sintering improves the reaction rate of the HMS phase. As previous studies have shown, the phase production in the ball-milling process can be partial or completely absent (depending on the ball-milling conditions), and the HMS phase can result from reactive sintering performed by hot press or SPS [60,62,65,70]. The hot-press sintering conditions used in this study, especially the temperature and duration, seem to be suitable, enabling a partial reactive sintering of the HMS phase. Ball milling was performed for 6 h, since the mechanochemical decomposition of HMS into MnSi begins to increase at longer times. This is in agreement with previous studies that have shown that the mechanochemical decomposition of the HMS phase is favored by prolonging the ball-milling time [61,64]. Tungsten carbide jar and balls were used rather than stainless steel since they have shown better results in terms of suppressing the MnSi phase [61]. Although the non-doped phase exhibits an almost single phase with slight traces of MnSi, MA increases the decomposition for the Al-doped phases. However, it must be noticed that the amount of MnSi still remains at low levels across the series Mn(Si_1−x_Al_x_)_1.75_ (0.02 ≤ x ≤ 0.05), without increasing with the increase in Al content. As a result, MnSi is not as destructive as shown later in the two recycled silicon-based cases, where MnSi exists in large amounts and causes a severe reduction in TE performance. For this reason, most of the Al-doped phases exhibit PF and ZT values close to those of the pristine phase or even slightly higher. Hole doping is accomplished by the substitution of Si by Al, since Al atoms possess one valence electron less than Si. A gradual increase in electrical conductivity is observed with the increase in Al content. The corresponding reduction in the Seebeck coefficient denotes the increase in hole concentration. It must be noticed here that the existence of small amounts of MnSi observed in Al-doped phases may also contribute to the increase in hole concentration, inducing a small effect in electrical transport properties, since MnSi is a metallic phase and exhibits a high number of holes as charge carriers [67,74]. The appreciable reduction in the Seebeck coefficient of Al-doped phases, especially at high temperatures, compared to that of the pristine compound, seems to confirm this suggestion. However, the rise in electrical conductivity overcomes the decrease in the Seebeck coefficient. Tuning the electrical transport properties, an increase in PF is achieved with the 2.5% and 4% Al-doped phases to exhibit higher values than those of the non-doped material. The analysis of thermal conductivity showed that there are no noticeable changes across the series Mn(Si_1−x_Al_x_)_1.75_ (0 ≤ x ≤ 0.05), apart from a slight increase in Al-doped phases, mainly due to the electronic contribution. This is explained by the fact that the determinant contribution in κ_tot_ comes from κ_lat_, which is not affected greatly by the Al substitution, since Al and Si have similar atomic masses. In addition, the low levels of MnSi are unable to produce a notable increase in the lattice thermal conductivity of the Al-doped phases. Finally, the 2.5% Al-doped material exhibits a maximum ZT of 0.43, which is one of the highest reported values for HMS phases developed exclusively by a dry ball-milling process [46,60,62,64,65].

On the other hand, the large amounts of metallic MnSi phase detected by powder XRD and SEM in the kerf-based phases are detrimental to the TE properties of these materials. MnSi is responsible for the abrupt decrease in the Seebeck coefficient, since it causes a strong increase in hole concentration in the kerf-based systems. The reduction in S is higher for the RST ODIN-0821-based product, which presents a higher percentage of MnSi phase than that of the RST 1-2-based counterpart. This results in a higher carrier density that is also confirmed by the higher electrical conductivity values. The presence of MnSi is also responsible for the increase in total lattice thermal conductivity of the two kerf-based systems, affecting both the electronic and lattice contribution. As expected, the increase in electronic contribution for the two recycled silicon-based materials follows the trend in electrical conductivity. However, the impact of the MnSi phase on the lattice thermal conductivity is much stronger, as shown in Figure 12c, increasing appreciably the values for the two kerf-based materials, compared with those of the Si-5N phase. This is in agreement with a previous study which has shown that MnSi exhibits a high κ_lat_, almost three times higher than that of HMS phases [74]. To extract information about the impact of the secondary phase on the lattice thermal conductivity, the effective volume fraction of each phase, HMS and MnSi, was calculated for the two kerf-based products using the Effective Medium Theory–Bruggeman approximation for composites and the corresponding lattice thermal conductivities of two phases. For the case of HMS, the obtained experimental value for high-purity Si-5N-based HMS was used, since it presents an almost single phase, while for the case of MnSi, the known κ_lat_ value at room temperature was used from the study of Cheng et al. [74]. The results are presented in the Supplementary Materials, Table S4. The RST ODIN-0821-based product presents a MnSi volume fraction of 18%, which is higher than that of its RST 1-2 counterpart (12%), since this sample exhibits the highest κ_lat_ values. This is also in accordance with the results from XRD refinements and backscattering SEM analysis, which showed the highest levels of MnSi for the RST ODIN-0821 case.

Comparing the two recycled silicon-based systems, it is obvious that the RST 1-2 case demonstrates better TE properties. This can be explained by the fact that the purification process is different for the two types of recycled silicon kerfs, with RST ODIN-0821 presenting a smaller amount of pure silicon. However, it must be clarified that the elemental impurities identified in the two types of kerf (Table S1 in Supplementary Materials) are not expected to affect the TE properties since they are at extremely low levels (in the order of a few hundred ppm). The deterioration in TE performance of the two kerfs comes clearly from the formation of MnSi in such large amounts. The RST ODIN-0821 case exhibits the lowest TE performance due to the highest levels of MnSi (ca. 38 wt.%). The reason seems to come from the fact that the silicon oxide layer (with a thickness of 2 nm), which was detected by STEM-EELS measurements on the surface of RST ODIN-0821 nanoflakes, may bind an appreciable amount of Si. This may change the actual stoichiometry of pure Si from the nominal value, favoring finally the formation of the MnSi phase. The EDX analysis (Figure S4 in Supplementary Materials) shows Si losses for the two kerf-based products. The overall results for silicon stoichiometry present a small reduction in at.%, compared with that of the Si-5N counterpart, with the RST ODIN-0821 case exhibiting the lowest value. It must be noted that the silicon oxide layer cannot be identified by the powder XRD measurements due to its amorphous phase, as our previous study also showed [13]. Another reason which may favor the mechanochemical decomposition to produce MnSi in such large amounts in both kerf cases is the fact that the two types of recycled silicon reagents are in nanoparticle form with an average size of 700 nm. Zhou et al. showed that the reduction in grain size that takes place during the ball-milling process enhances the formation of MnSi [61]. In addition, this study concluded that the mechanochemical decomposition seems to be correlated with the anisotropic structural properties of the HMS phases. It is noted here that the NCL tetragonal superstructures can be expanded along the *z* axis, reaching a height of several nanometers. The Rietveld analysis showed that the developed HMS phases are best described by the Mn_15_Si_26_ superstructure, which has a *c* lattice parameter of ca. 6.5 nanometers (Table S3 in Supplementary Materials). Interestingly, in our previous study it was shown that both types of recycled silicon nanopowders exhibit a highly anisotropic flake-like shape with a thickness only few tens of nanometers. This may affect significantly the mechanochemical reaction of manganese with silicon nanoflakes, impeding the formation of the HMS phase and consequently favoring the composition of MnSi. Finally, the development of new RST 1-2-based products was also investigated by reducing the ball-milling time, in an attempt to suppress the amount of the MnSi phase. In the Supplementary Materials, Figure S5 shows that the reduction in ball-milling time to 2 h did not have a positive effect in eliminating the secondary phase, enhancing our suggestion that the morphology of Si kerf may be responsible for the formation of MnSi. More investigations need to be carried out in the future that focus on the suppression of MnSi in the recycled-based phases, with the aim of improving their TE figure-of-merit. Changing the ball-milling conditions such as the BPR or introducing organic solvents as milling media may favor the formation of HMS instead of the metallic MnSi phase. The addition of Si kerf excess should also be attempted in an effort to counterbalance the lack of pure silicon in the recycled byproduct.

## 5. Conclusions

HMS TE materials of the general formula Mn(Si_1−x_Al_x_)_1.75_ (0 ≤ x ≤ 0.05) are developed by MA and hot-press sintering. Powder XRD validates the desired HMS phase across the series, while small amounts of MnSi are also identified, especially for the Al-doped products. Backscattering SEM imaging and EDX analysis confirm the existence of the secondary phase in the microstructure of the developed phases. Hole doping is carried out through Al substitution to tune the electrical transport properties in the series. An appreciable increase in electrical conductivity is observed with the increase in Al content, while the corresponding decrease in the Seebeck coefficient denotes the increase in carrier density. An improvement in PF is achieved for the Mn(Si_1−x_Al_x_)_1.75_ (0 ≤ x ≤ 0.05) series, with the 4% Al-doped phase exhibiting a maximum value of ca. 14.5 μW cm^−1^ K^−2^ at 725 K. The analysis of thermal conductivity shows that Al substitution does not cause any significant effect on the lattice contribution, since no distinctive changes are observed across the series. Despite the existence of the MnSi phase at low levels, an increase in TE figure-of-merit is accomplished for the Al-doped phases. The HMS phase with 2.5% Al content presents the best TE performance with a maximum ZT of 0.43 at 773 K.

In addition, 2.5% Al-doped phases are developed by the MA method using two types of recycled silicon based on kerf byproducts, which come from PV manufacturing. Powder XRD measurements for the two recycled silicon-based products show the desired HMS pattern and the existence of the MnSi phase in relatively large amounts. The high levels of MnSi cause an obvious reduction in the Seebeck coefficient, due to the increase in the system’s carrier density. This together with an increase in thermal conductivity results in a noticeable reduction in TE performance in comparison with that of the Si-5N counterpart. Silicon losses seem to be responsible for the formation of the MnSi phase, while the size and shape of recycled silicon reagent nanoflakes may also affect the mechanochemical reaction, impeding the formation of asymmetric HMS structures and finally favoring the formation of the MnSi phase. However, between the two recycled silicon-based products, the RST 1-2 case presents lower levels of the MnSi phase and as a result, it exhibits better TE properties, reaching a maximum ZT of 0.18 at 773 K. New research directions should be investigated in the future, aiming to suppress the metallic MnSi phase in both kerf-based products and enhancing their TE performance.

## Data Availability

The data presented in this study are available on request from the corresponding author due to privacy reasons.

## Supplementary Materials

The following supporting information can be downloaded at: https://www.mdpi.com/article/10.3390/nano15161286/s1. Figure S1: (a) Powder XRD patterns of pristine phase MnSi_1.75_ ball milled for 3, 6, and 9 h. Reflection position for the secondary phase, MnSi, is marked with asterisk. (b) The characteristic peak of MnSi close to 44.3^o^ for the three different ball milling times; Figure S2: EDX measurements performed in (a) the dark grey matrix and (b) light grey spots of Si-5N-based pristine phase (x = 0); Figure S3: Powder XRD Rietveld refinements for the 2.5% Al doped phases based on (a) high-purity Si (Si-5N), (b) RST 1-2 Si kerf and (c) RST ODIN-0821 Si kerf. Observed (black crosses), refined (red solid lines) and difference (blue bottom line) profiles. Reflection positions of the HMS Mn_15_Si_26_ phase are indicated by olive markers, while MnSi is indicated by navy markers; Figure S4: Overall EDX measurements for the 2.5% Al doped phases based on (a) high-purity Si (Si-5N), (b) RST 1-2 Si kerf and (c) RST ODIN-0821 Si kerf; Figure S5: Powder XRD patterns of RST 1-2 based-product ball milled for 2, 3, 4, and 6 h; Table S1: Elemental impurities of two types of recycled silicon based on Si kerf from PV manufacturing; Table S2: Quantification results of phase fraction of 2.5 % Al doped products extracted by Rietveld refinements; Table S3: Refined lattice parameters of HMS phase, Mn_15_Si_26_, with space group I$\bar{4}2d$; Table S4: Calculations of volume fraction of two phases, HMS and MnSi, acting on lattice thermal conductivity.
