# Supplementary material for: Nanostructured Higher Manganese Silicide Thermoelectrics Developed by Mechanical Alloying Using High-Purity and Recycled Silicon
Source: Nanomaterials (Basel). 2025 Aug 21;15(16):1286. doi: 10.3390/nano15161286 (PMC12388703; doi:10.3390/nano15161286)
Supplement: Supplementary file 1 [file nanomaterials-15-01286-s001.zip › nanomaterials-3783863-Supplementary Materials.pdf]

nanomaterials

MDPI

Supplementary Material

Nanostructured Higher Manganese Silicide Thermoelectrics developed by Mechanical Alloying using High-purity and Recycled Silicon

Panagiotis Mangelis [1,*], Kostas Georgiou [1], Panagiotis Savva Ioannou [1], Savvas Hadjipanteli [1], Anne-Karin Søiland [2] and Theodora Kyratsi [1,*]

[1] Department of Mechanical and Manufacturing Engineering, University of Cyprus, Nicosia, 1678, Cyprus; georgiou.kostas@ucy.ac.cy (K.G.); ioannou.s.panagiotis@ucy.ac.cy (P.S.I.); hadjipanteli.savvas@ucy.ac.cy (S.H.)

[2] ReSiTec AS, Setesdalsveien 110, Kristiansand, 4617, Norway; aks@resitec.no

* Correspondence: mangelis.panagiotis@ucy.ac.cy (P.M.), kyratsi.theodora@ucy.ac.cy (T.K.)

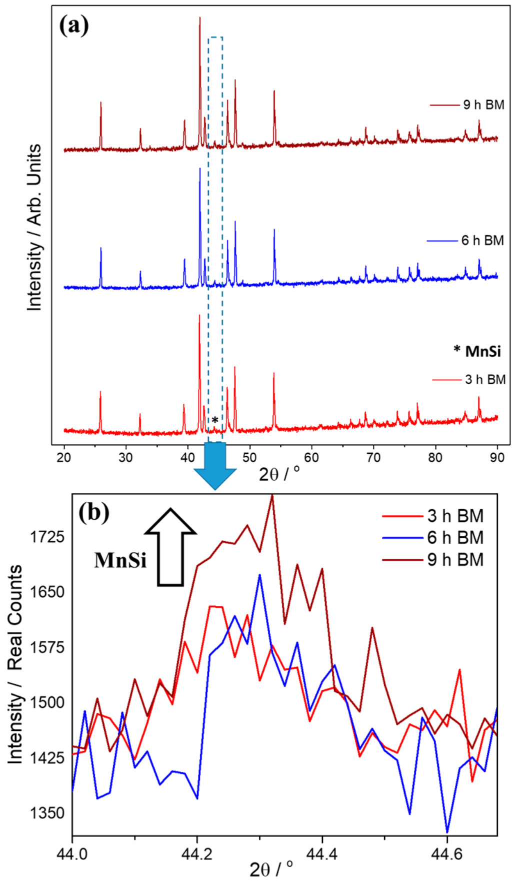

Figure S1. (**a**) Powder XRD patterns of pristine phase $MnSi_{1.75}$ ball milled for 3, 6 and 9 h. Reflection position for the secondary phase, MnSi, is marked with asterisk. (**b**) The characteristic peak of MnSi close to 44.3 ° for the three different ball milling times.

Academic Editor: Keqiu Chen

Received: 12 July 2025
Revised: 11 August 2025
Accepted: 19 August 2025
Published: 21 August 2025

Citation: To be added by editorial staff during production.

Copyright: © 2025 by the authors. Submitted for possible open access publication under the terms and conditions of the Creative Commons Attribution (CC BY) license (https://creativecommons.org/licenses/by/4.0/).

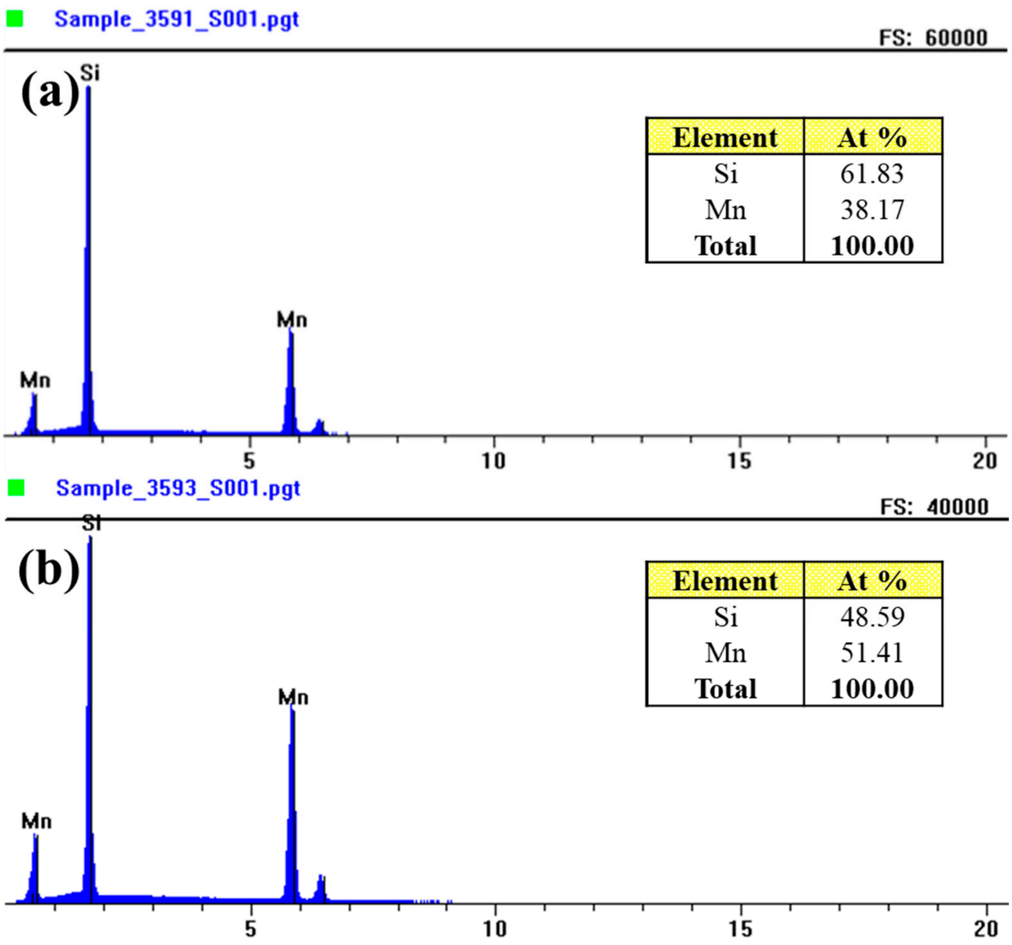

Figure S2. EDX measurements performed in (**a**) the dark grey matrix and (**b**) light grey spots of Si-5N-based pristine phase (x = 0).

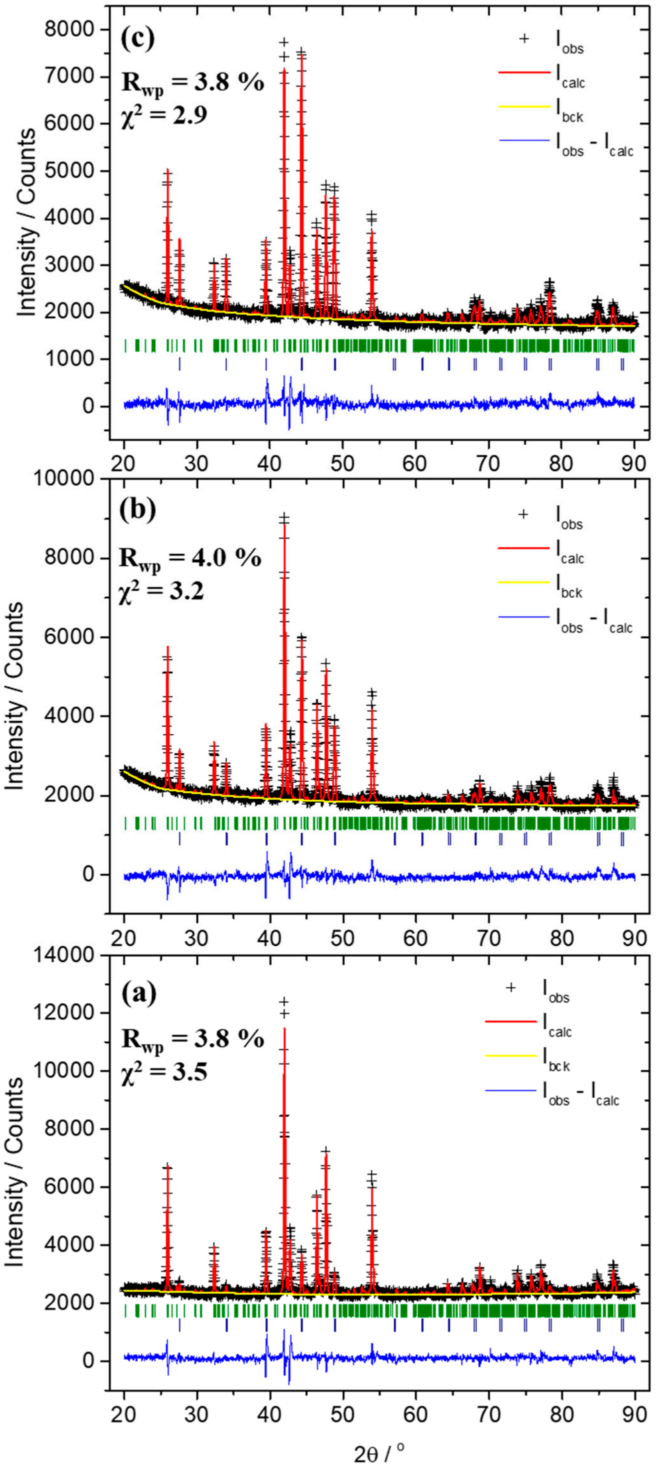

Figure S3. Powder XRD Rietveld refinements for the 2.5% Al doped phases based on (**a**) high-purity Si (Si-5N), (**b**) RST 1-2 Si kerf and (**c**) RST ODIN-0821 Si kerf. Observed (black crosses), refined (red solid lines) and difference (blue bottom line) profiles. Reflection positions of the HMS $Mn_{15}Si_{26}$ phase are indicated by olive markers, while MnSi is indicated by navy markers.

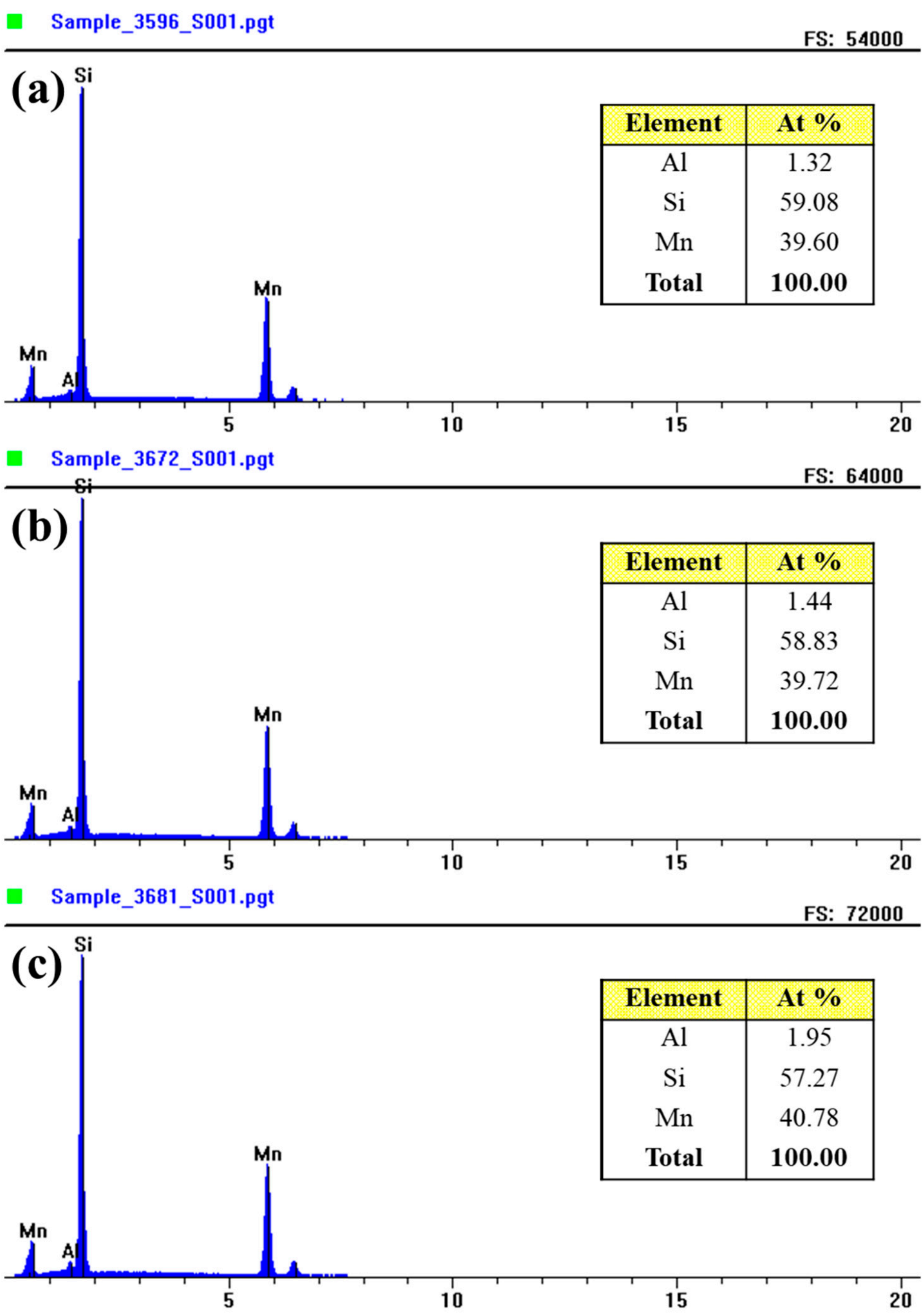

Figure S4. Overall EDX measurements for the 2.5% Al doped phases based on (**a**) high-purity Si (Si-5N), (**b**) RST 1-2 Si kerf and (**c**) RST ODIN-0821 Si kerf.

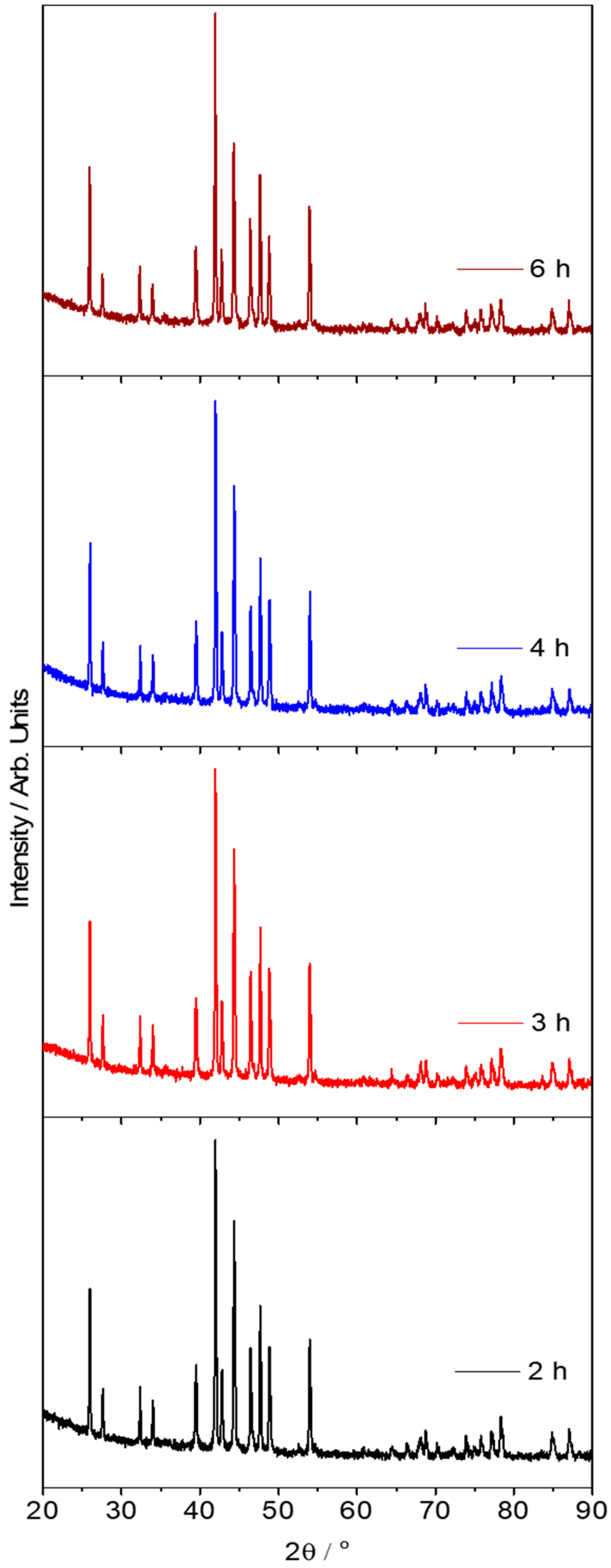

Figure S5. Powder XRD patterns of RST 1-2 based-product ball milled for 2, 3, 4, and 6 h.

Table S1. Elemental impurities of two types of recycled silicon based on Si kerf from PV manufacturing.

Recycled Si Type	Al (from Al_2O_3) / ppm	Ca / ppm	Ni / ppm	Ga / ppm	Fe, B, P / ppm
RST 1-2	30	560	≈ 25	< 5	< 5
RST ODIN-0821	7000	460	≈ 135	68	< 5

Table S2. Quantification results of phase fraction of 2.5% Al doped products extracted by Rietveld refinements.

Sample	$Mn_{15}Si_{26}$ / wt. %	MnSi / wt. %
**Si-5N**	93.5(1)	6.5(1)
**RST 1-2**	78.8(1)	21.2(1)
**RST ODIN-0821**	61.9(1)	38.1(2)

Table S3. Refined lattice parameters of HMS phase, $Mn_{15}Si_{26}$, with space group $I\bar{4}2d$.

Sample	a / Å	c / Å	V / Å^3
**Si-5N**	5.5303(1)	65.537(2)	2004.39(5)
**RST 1-2**	5.5284(1)	65.512(2)	2002.26(7)
**RST ODIN-0821**	5.5282(1)	65.515(2)	2002.25(8)

Table S4. Calculations of volume fraction of two phases, HMS and MnSi, acting on lattice thermal conductivity.

Sample	Volume fraction of HMS	Volume fraction of MnSi
**RST 1-2**	88%	12%
**RST ODIN-0821**	82%	18%
